# Advances in glycolysis research in gastric cancer: molecular mechanisms, regulatory networks, and therapeutic potential

**DOI:** 10.3389/fonc.2025.1678681

**Published:** 2025-10-27

**Authors:** Jungang Dong, Weiyan Li, Liang Ma, Jinrong Yang, Decheng Gan, Haixia Xue, Li Pu, Lili Zhang, Kelan Zhang, Yonglong Jia, Qingyu Ma

**Affiliations:** ^1^ Department of Classical Chinese Medicine, Gansu Provincial Hospital of Traditional Chinese Medicine, Lanzhou, Gansu, China; ^2^ Department of Critical Care Medicine, Gansu Provincial Hospital of Traditional Chinese Medicine, Lanzhou, Gansu, China

**Keywords:** gastric cancer, glycolysis, Warburg effect, non-coding RNA, tumor metabolism, therapeutic targets

## Abstract

Glycolysis is a central metabolic pathway in cancer cells, contributing significantly to the initiation, progression, and therapeutic resistance of gastric cancer. Advances in molecular biology and metabolomics have clarified the regulatory landscape of glycolysis, particularly its interactions with the tumor microenvironment and key signaling pathways. However, important gaps remain in understanding the precise functions and interactions of key regulatory factors. This review presents an overview of recent progress in glycolysis research in gastric cancer, focusing on essential regulators such as CENPU, CD73, SALL4, and MAOA, non-coding RNAs (e.g., circRNAs, lncRNAs, and miRNAs), and exosome-mediated metabolic reprogramming driven by tumor-associated macrophages. It also discusses the prognostic value of glycolysis-related genes and their potential as therapeutic targets, including the application of natural compounds and small-molecule inhibitors in anti-glycolytic strategies. These findings provide valuable insights into the metabolic mechanisms underlying gastric cancer and highlight the potential for developing metabolism-targeted therapies.

## 1 Introduction

Gastric cancer is a globally prevalent malignancy with a high mortality rate, making its molecular pathogenesis and therapeutic strategies essential areas of investigation (1). Despite advances in diagnostic and treatment approaches, the persistently rising mortality highlights the need for deeper mechanistic insights (2). Glycolysis has become a central feature of cancer cell metabolic reprogramming. Even under normoxic conditions, cancer cells preferentially rely on glycolysis for energy production, a phenomenon known as the Warburg effect (3). This metabolic shift supports tumor proliferation, invasion, and therapeutic resistance, underscoring the clinical importance of glycolytic regulation in gastric cancer.

Recent studies have identified several molecular regulators of glycolysis in gastric cancer. For example, Centromere Protein U (CENPU) enhances glycolysis and proliferation by upregulating High Mobility Group Box 2 (HMGB2) (4), while CD73 is induced under hypoxia to boost glycolytic capacity and promote tumor growth (5). These findings provide new insights into metabolic reprogramming and suggest promising therapeutic targets.

Non-coding RNAs are also key regulators of glycolysis. Long non-coding RNA SNHG7 modulates miR-34a to regulate glycolytic enzymes, influencing chemoresistance and proliferation (6). Similarly, circular RNAs such as circ_0067514 regulate glycolysis via the miR-654-3p/LATS2 axis (7). These findings reveal a complex RNA-based regulatory network that highlights the biological complexity of glycolysis in gastric cancer.

**Figure 1:**
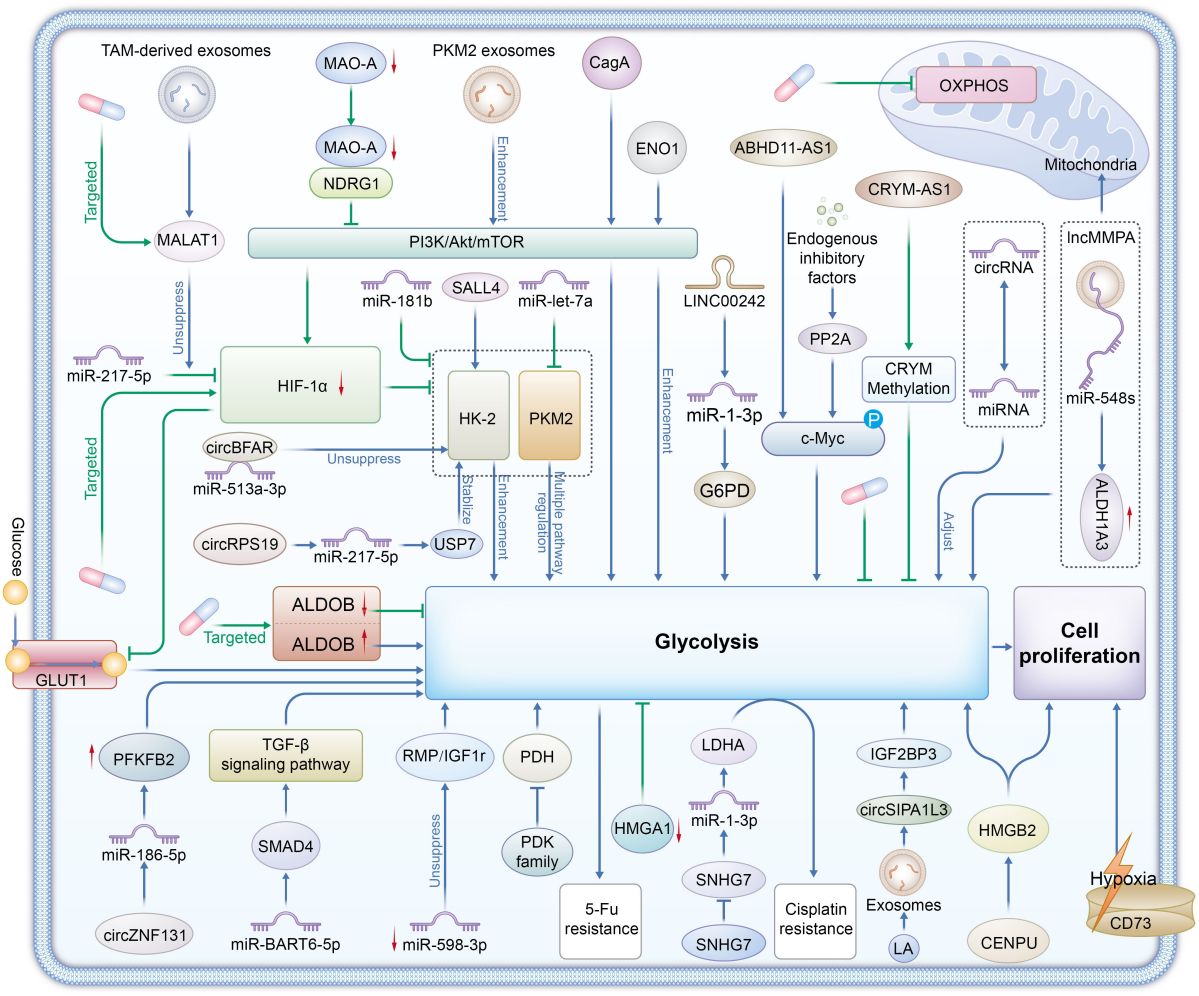
Enzymes Glycolytic enzymes: HK2 (Hexokinase 2), PKM2 (Pyruvate kinase M2), LDHA (Lactate dehydrogenase A), ALDOB (Aldolase B), and the PDK family (Pyruvate dehydrogenase kinases) are central to glycolysis regulation in gastric cancer. Signaling Pathways PI3K/Akt/mTOR, TGF-β, HIF-1α, and c-Myc signaling pathways regulate glycolytic enzymes, promoting glycolysis even under hypoxic conditions, which is a hallmark of the Warburg effect in tumors.CD73 contributes to adenosine production, enhancing glycolysis and promoting tumor cell survival and immune evasion. Non-Coding RNAs LncRNAs (e.g., SNHG7, ABHD11-AS1), circRNAs (e.g., circBFAR, circZNF131), and miRNAs (e.g., miR-181b, miR-let-7a) regulate the expression and stability of glycolytic enzymes. They facilitate metabolic reprogramming, enhance chemoresistance, and support tumor progression by modulating glycolysis. Exosome-Mediated Metabolic Reprogramming Exosomes from TAMs (Tumor-Associated Macrophages) and gastric cancer cells (carrying MALAT1, PKM2, circSIPA1L3) transfer metabolic regulators between cells, promoting glycolysis and lactate production, further supporting tumor growth and metastasis. Therapeutic Targets Glycolytic enzymes (e.g., HK2, LDHA), non-coding RNAs (e.g., MALAT1, SNHG7), and signaling pathways (e.g., PI3K/Akt/mTOR, HIF-1α) are potential therapeutic targets for overcoming chemoresistance and inhibiting gastric cancer progression. Targeting these molecules could disrupt the Warburg effect and reduce tumor cell proliferation.

In summary, understanding the molecular regulation of glycolysis in gastric cancer is essential for developing novel therapeutic strategies(As shown in **Figure 1**). Targeting glycolytic pathways presents new opportunities for intervention and may improve clinical outcomes for patients with gastric cancer.

## 2 Molecular mechanisms regulating glycolysis in gastric cancer

Glycolysis is essential for the metabolic reprogramming of gastric cancer cells, particularly under the “Warburg effect,” where cancer cells preferentially rely on glycolysis to generate energy and metabolic intermediates, even in an oxygen-rich environment. This metabolic shift not only supplies the energy required for rapid tumor growth but is also closely associated with tumor invasion, metastasis, and resistance to treatment. Therefore, a comprehensive investigation into the molecular mechanisms underlying glycolysis in gastric cancer is crucial for uncovering the malignancy-related characteristics of the disease and for identifying potential therapeutic targets.

Recent studies have highlighted the roles of key enzymes (e.g., HK2 and PKM2) and signaling pathways (e.g., PI3K/Akt/mTOR, c-Myc, HIF-1α) in gastric cancer metabolism. However, the intricate network of glycolytic regulation, particularly the involvement of non-coding RNAs (such as miRNAs and lncRNAs), the impact of the tumor microenvironment, and the interactions between metabolic and signaling pathways, remain underexplored. Investigating these mechanisms will enhance the understanding of metabolic regulation in gastric cancer and offers substantial potential for personalized therapy and the development of novel anti-cancer approaches.

### 2.1 Key proteins and enzymes

CENPU (Centromere Protein U) is pivotal for gastric cancer development (As shown in **Table 1**). CENPU stabilizes chromosomes through its involvement in the cell cycle and regulates glycolytic enzyme expression, including HK2, LDHA, and PKM2, via activation of the PI3K/Akt signaling pathway. This mechanism supplies energy to cancer cells for rapid proliferation and migration (8). Activation of PI3K/Akt increases the synthesis of these enzymes, allowing cancer cells to obtain energy quickly through anaerobic glycolysis, even in hypoxic conditions. Additionally, CENPU facilitates energy provision during cell division by closely coordinating with the cell cycle, enabling tumor cells to acquire more metabolic intermediates during proliferation, thus advancing gastric cancer development (4).

**Table 1:** Key proteins and enzymes regulating glycolysis in gastric cancer.

Molecule/protein	Main mechanism of action	Glycolytic targets	Function in gastric cancer	References
CENPU	Activates PI3K/Akt pathway, promotes expression of HK2, LDHA, and PKM2, maintaining energy supply during cell cycle	HK2, LDHA, PKM2	Promotes proliferation, survival, and enhanced metabolism in gastric cancer cells under hypoxic conditions	4, 8
HMGB2	Enhances HIF-1α activity, upregulates LDHA, PKM2, HK2; activates NF-κB to enhance glycolysis	LDHA, PKM2, HK2	Facilitates hypoxic adaptation, growth, and drug resistance	9, 10
CD73	Regulates expression of HK2, PKM2, LDHA through cAMP/PKA pathway via adenosine-A2A/A2B receptors	HK2, PKM2, LDHA	Enhances glycolysis, immune evasion, and invasion in gastric cancer	11–13
SALL4	Regulates HK2 to enhance glycolysis	HK2	Promotes glycolysis and tumor progression in gastric cancer cells	14, 15
MAO-A	Regulates mitochondrial function and glycolysis	Not specifically listed	Suppresses proliferation and metastasis, low expression promotes tumor progression	14, 15
ALDOB	Low expression releases inhibition of AKT pathway, enhancing glycolysis	HK2, AKT	High expression inhibits glycolysis and increases chemotherapy sensitivity	16
PKM2	Upregulates lactate generation genes, nuclear translocation regulates STAT3/c-Myc, anti-apoptotic, extracellular vesicle remodeling	LDHA, GLUT1, HIF-1α	Promotes glycolysis, invasion, drug resistance, and immune regulation	17–21
PDK Family	Phosphorylates PDH, blocks pyruvate to acetyl-CoA conversion, forces lactate production	HK2, LDH	Enhances glycolysis, reduces oxidative phosphorylation, maintains low ROS environment	22–27
ENO1	Catalyzes glycerol phosphate to phosphoenolpyruvate, activates AKT/EMT signaling, HSP90 mediates enzyme complex formation	LDHA, PGK1, PKM2	Enhances glycolytic flux, promotes drug resistance and stemness characteristics	28–30
GLUT1/4	Increases glucose uptake, driving glycolytic metabolic flux	HK2, LDHA	Overexpression promotes proliferation, drug resistance, and prevents cell death	31–34

HMGB2 (High Mobility Group Box 2) is a non-histone chromatin-binding protein involved in several cellular processes. In gastric cancer, overexpression of HMGB2 drives glycolysis through two main signaling pathways. First, HMGB2 boosts the transcriptional activity of HIF-1α, leading to upregulation of glycolytic enzymes such as LDHA, PKM2, and HK2, which help gastric cancer cells sustain glycolysis under hypoxic conditions (9). Second, HMGB2 activates the NF-κB signaling pathway, which increases glycolytic efficiency and provides energy for tumor cell proliferation and survival (10). Furthermore, HMGB2 overexpression contributes to chemoresistance in gastric cancer by enhancing energy production through glycolysis, enabling cells to survive chemotherapy or radiation stress.

CD73 (Adenosine 5’-nucleotidase) is an enzyme on the cell membrane that converts adenosine monophosphate to adenosine. Adenosine, an important signaling molecule, regulates glycolysis, immune evasion, and tumor metastasis in gastric cancer. First, adenosine binds to A2A and A2B receptors, activating the cAMP/PKA pathway and increasing intracellular cAMP levels, which then regulate the expression of glycolytic enzymes HK2, PKM2, and LDHA through PKA, enhancing glucose uptake, lactate production, and metabolic activity to support cancer cell proliferation (11). Second, adenosine production suppresses T cell and NK cell function via the A2A receptor, promoting immune evasion and allowing gastric cancer cells to escape immune system attacks (12). Through its dual action on both A2A and A2B receptors, adenosine not only suppresses immune responses but also increases gastric cancer cell motility and invasiveness, facilitating tumor cell migration and dissemination (13). Consequently, high expression of CD73 boosts the metabolic activity of gastric cancer cells and supports immune suppression and invasion, allowing tumor cells to grow and metastasize in hostile microenvironments, making CD73 a potential therapeutic target for gastric cancer.

SALL4 drives gastric cancer progression by regulating hexokinase II (HK2), further highlighting the role of glycolytic enzymes in tumor metabolism. Monoamine oxidase A (MAO-A) modulates mitochondrial function and glycolysis, inhibiting gastric cancer cell proliferation and metastasis (14, 15).

ALDOB, a key glycolytic enzyme, functions as a tumor suppressor in gastric cancer. Low ALDOB expression enhances glycolytic activity, contributing to tumor progression. Specifically, reduced ALDOB expression in gastric cancer tissues activates downstream glycolytic enzymes by lifting inhibition on the AKT signaling pathway, thereby promoting aerobic glycolysis, tumor growth, and metastasis. Overexpression of ALDOB, however, inhibits glycolysis and increases chemosensitivity (16), suggesting its potential as a therapeutic target.

Hexokinase II (HK2), which catalyzes the phosphorylation of glucose to glucose-6-phosphate, directly regulates glycolytic flux. Increased HK2 activity significantly enhances glucose uptake, lactate production, and extracellular acidification rate (35, 36), while simultaneously reducing oxygen consumption to support aerobic glycolysis (37). HK2 also interacts with PDLIM1, activating the Wnt/β-catenin pathway (38) and, together with HIF-1α, upregulates glycolytic enzymes such as PFK2 and LDHA (39), forming a positive feedback loop that sustains elevated glycolytic flux.

Pyruvate kinase M2 (PKM2) coordinates both glycolytic metabolism and malignant phenotypes in gastric cancer via multiple downstream pathways. As a rate-limiting enzyme, PKM2 catalyzes the conversion of phosphoenolpyruvate (PEP) to pyruvate and promotes the transcription of LDHA and GLUT1, thereby enhancing lactate production and glucose uptake (17). PKM2 also translocates to the nucleus, where it phosphorylates STAT3 and forms a complex with c-Myc, upregulating HIF-1α and EMT-related genes such as Snail and Twist, driving invasion and glycolytic reprogramming (18). Moreover, PKM2 maintains cell survival by stabilizing NF-κB p65 and upregulating Bcl-xL, thus inhibiting mitochondrial apoptosis (19). PKM2 is secreted via exosomes, taken up by tumor-associated macrophages (TAMs), promoting M2 polarization and a glycolysis-enhancing microenvironment (21). Of note, the newly identified PRDX2/PKM2/STAT3 positive feedback loop amplifies glycolysis: nuclear PKM2 activates STAT3, which upregulates PRDX2, and PRDX2 inhibits PKM2 ubiquitination, stabilizing PKM2 levels (20).

The PDK family inhibits the pyruvate dehydrogenase (PDH) E1α subunit via phosphorylation, thereby blocking the conversion of pyruvate to acetyl-CoA and redirecting pyruvate toward lactate production by LDH (22, 23). PDK2 stabilizes HIF-1α, upregulating glycolytic enzymes (24), and PDK3 enters the nucleus to form a feedback loop with HSF1, reinforcing the expression of HK and LDH. PDK4-mediated PDH inhibition also reduces mitochondrial ROS generation (25), maintaining a low-ROS environment to support sustained glycolysis (26, 27).

Enolase 1 (ENO1) catalyzes the conversion of 2-phosphoglycerate to phosphoenolpyruvate and is highly expressed in gastric cancer, enhancing glycolytic flux and contributing to chemoresistance (28). ENO1 also activates the AKT signaling pathway, promoting EMT and enhancing proliferation, migration, and invasion (29). Together with PGK1 and PKM2, ENO1 forms the HGEO multi-enzyme complex, which, under HSP90 mediation, boosts nuclear glycolytic output and lactate production, fostering stem-like features and resistance traits in tumor cells (30).

Glucose transporters (GLUTs) are central to glycolysis in gastric cancer by promoting glucose uptake and supporting energy metabolism, proliferation, and survival. Overexpression of GLUT1 or GLUT4 enhances glucose influx and the activity of glycolytic enzymes such as HK2 and LDHA, leading to increased lactate accumulation and ATP production, thus promoting growth and suppressing apoptosis (31, 32). Inhibition of GLUT1 reduces glucose consumption and lactate production and decreases ATP levels, directly suppressing glycolysis and inducing cancer cell death (33). Clinically, GLUT1 overexpression correlates with glycolytic phenotypes characterized by upregulation of HK2, PKM2, and LDHA, and predicts resistance to chemotherapy such as 5-fluorouracil and ramucirumab (34). Therefore, GLUTs are critical mediators of energy metabolism and malignancy in gastric cancer.

Lactate dehydrogenase (LDH), which catalyzes the terminal step of glycolysis by converting pyruvate to lactate and regenerating NAD^+^, is highly expressed in gastric cancer (40). Inhibition of this process—e.g., via β-sitosterol—impairs NAD^+^ regeneration, disrupts glycolysis, and induces apoptotic signaling such as mitochondrial depolarization and caspase activation (41). Studies on these key enzymes and proteins provide a critical molecular basis for understanding the glycolytic phenotype in gastric cancer and offer promising targets for therapeutic development.

### 2.2 Core signaling pathways

The PI3K/Akt/mTOR signaling pathway plays a central role in the regulation of glycolysis(As shown in **Table 2**). In gastric cancer, MAOA (monoamine oxidase A) is generally downregulated, but its re-expression has been shown to suppress the PI3K/Akt/mTOR pathway via interaction with NDRG1. This inhibition leads to reduced activity of the downstream transcription factor HIF-1α, thereby decreasing the expression and function of key glycolytic regulators including GLUT1, HK2, PFK1, PKM2, and LDHA. The result is a significant reduction in glucose uptake, extracellular acidification rate (ECAR), and lactate production, while mitochondrial oxidative metabolism is enhanced, ultimately suppressing cell proliferation and migration and promoting apoptosis (45). Similar inhibitory effects on this pathway have been demonstrated in other gastric cancer models, such as Bufalin treatment (46, 47), and in related tumor studies involving TRIM59 suppression (48) and ILF3 overexpression (49), further reinforcing the critical role of the PI3K/Akt/mTOR axis in maintaining aberrant tumor metabolism.

**Table 2:** Metabolic signaling pathways regulating glycolysis in gastric cancer.

Signaling pathway	Upstream factors	Downstream targets	Mechanism of action	Functional impact	References
PI3K/Akt/mTOR	CENPU, CagA, EGFR	HK2, PKM2, LDHA	Activates glycolytic enzyme expression, enhances glucose uptake and lactate production	Promotes tumor proliferation and drug resistance	4, 8, 42, 45
HIF-1α Pathway	Hypoxia, ROS	HK2, LDHA, GLUT1	Hypoxia induces HIF-1α transcription, enhancing glycolytic enzyme expression	Facilitates survival in hypoxic conditions, metabolic reprogramming	24, 39
c-Myc Pathway	PKM2 Nuclear Translocation, STAT3	LDHA, HK2	Transcriptionally regulates glycolytic enzyme expression, drives Warburg effect	Promotes proliferation, invasion, and tumor stemness	17, 18
NF-κB Pathway	HMGB2, Inflammatory Factors	PKM2, LDHA	Activates transcription, promotes glycolysis and tumor cell survival	Enhances drug resistance and anti-apoptotic activity	10, 20
Wnt/β-catenin Pathway	HK2 and PDLIM1 Interaction	PFK2, LDHA	Upregulates glycolytic enzyme expression, enhances glycolytic flux	Promotes metabolic reprogramming and tumor invasion	38, 43, 44

Protein phosphatase 2A (PP2A), a well-established tumor suppressor, is functionally impaired in gastric cancer due to the overexpression of endogenous inhibitors such as SET and CIP2A. This leads to sustained phosphorylation of c-Myc at Ser62, which in turn upregulates key glycolytic enzymes such as GLUT1, HK2, and LDHA. The result is enhanced glucose uptake, lactate production, and activation of the Warburg effect, ultimately promoting tumor growth (50). Another study revealed that high expression of HMGA1 in gastric cancer patients positively correlates with c-Myc levels and glycolytic markers like GLUT1 and HK2. Silencing HMGA1 significantly reduces glucose uptake, lactate production, and ECAR, while inhibiting cell proliferation and invasion—highlighting the pivotal role of c-Myc in driving glycolysis and supporting the therapeutic potential of PP2A-mediated c-Myc regulation (51). These findings suggest that PP2A affects gastric cancer metabolism by modulating c-Myc, offering novel targets for intervention.

Infection with *Helicobacter pylori*, particularly through its virulence factor CagA, further activates the PI3K/Akt pathway upon translocation into gastric cancer cells, thereby enhancing aerobic glycolysis (52, 53). Inhibiting Akt (e.g., with LY294002) or blocking glycolysis (e.g., with 2-deoxy-D-glucose or oxamate) completely reverses CagA-induced 5-FU resistance and restores chemosensitivity in both *in vitro* and *in vivo* models (54). These findings reveal a pathway by which CagA promotes glycolysis via Akt activation, thereby contributing to chemoresistance in gastric cancer.

In summary, the interplay between metabolic and signaling pathways plays a critical role in the development and progression of gastric cancer. Key regulators such as the PI3K/Akt/mTOR signaling axis, PP2A, and *H. pylori* CagA protein not only deepen our understanding of metabolic reprogramming in tumors but also provide promising therapeutic targets. These findings underscore the complex interdependence of metabolic and signaling networks and highlight the need to consider both in the study of cancer biology.

### 2.3 Non-coding RNAs in glycolysis

In the metabolic reprogramming of gastric cancer, miRNA, lncRNA, and circRNA interact to regulate the glycolytic pathway, influencing the energy metabolism of tumor cells (As shown in **Table 3**). miRNA modulates glycolysis by binding to the 3’ UTR of glycolysis-related genes (such as HK2, LDHA, and PKM2), suppressing their expression. However, certain lncRNAs (such as H19 and MALAT1) counteract the inhibition of glycolytic enzymes by binding to miRNA or competitively interacting with it, thereby promoting the expression of these enzymes and further enhancing the glycolytic process. For instance, H19 competes with miR-29, suppressing the miRNA function and subsequently upregulating the expression of PKM2 and LDHA, which intensifies the glycolytic response (46). Meanwhile, circRNA (such as circ_0001445) competes with miRNA (such as miR-145), alleviating the inhibitory effect on glycolytic enzymes, thereby promoting the expression of LDHA and PKM2 (56). This circRNA-miRNA interaction mechanism helps gastric cancer cells sustain a high metabolic rate, supporting tumor cell proliferation and growth. Notably, similar non-coding RNA regulatory mechanisms have been observed not only in gastric cancer but also in other tumor types, such as breast cancer, lung cancer, and colon cancer (56). This suggests that the interplay between non-coding RNAs is crucial in the metabolic regulation of various cancers.

**Table 3:** Non-coding RNAs regulating glycolysis in gastric cancer.

RNA Type	Specific molecule	Regulated targets	Regulatory mechanism	Function in gastric cancer	References
lncRNA	H19, MALAT1	PKM2, LDHA	Competes with miRNA, relieves suppression on glycolytic enzyme expression, promoting glycolysis	Enhances glycolysis, promotes tumor proliferation and drug resistance	46
lncRNA	LINC00242	G6PD	Promotes glycolysis and gastric cancer cell proliferation through the miR-1-3p/G6PD axis	Promotes glycolysis and gastric cancer cell proliferation	42
lncRNA	SNHG7	LDHA	Inhibits miR-34a, regulates LDHA expression, and mediates chemotherapy resistance	Increases cisplatin resistance and promotes glycolysis and cell survival	6
lncRNA	ABHD11-AS1	c-Myc	Upregulates c-Myc, promoting glycolysis and tumor progression	Promotes cell proliferation and metabolism, driving tumor progression	55
lncRNA	CRYM-AS1	CRYM	Binds to EZH2, regulates CRYM methylation, inhibits glycolysis and gastric cancer cell proliferation	Reduces glycolysis and proliferation, potentially a therapeutic target	43
circRNA	circ_0001445	LDHA, PKM2	Competes with miR-145, relieving inhibition on glycolytic enzymes	Promotes glycolysis, enhances gastric cancer cell proliferation and drug resistance	56
circRNA	circRPS19	HK2	Mediates HK2 protein deubiquitination via the miR-125a-5p/USP7 axis, stabilizing its enzyme activity	Enhances glycolysis, increases lactate production and glucose uptake	57
circRNA	circZNF131	PFKFB2	Upregulates PFKFB2 expression by binding to miR-186-5p, activating the phosphofructokinase pathway	Enhances glycolysis, increases glycolytic flux	58
circRNA	circUBE2Q2	LDHA	Releases STAT3 signaling through competitive binding with miR-370-3p, promoting transcription of glycolytic genes	Enhances lactate production, promotes tumor progression	59
circRNA	circ_0006089	GLUT1	Increases GLUT1 expression through the miR-361-3p/TGFB1 axis, promoting glucose uptake	Increases glucose uptake and lactate production, supporting gastric cancer cell proliferation	60
miRNA	miR-181b	HK2	Binds to HK2 3′-UTR, suppresses its expression, reducing glucose uptake and lactate production	Inhibits glycolysis, reduces tumor cell metabolic activity	36
miRNA	miR-let-7a	PKM2	Targets PKM2, downregulates its expression, inhibiting glycolytic flux	Inhibits glycolytic flux, reduces lactate production	61
miRNA	miR-139-5p	PRKAA1	Regulates AMPKα1, affecting energy metabolism pathways	Modulates energy metabolism, affecting glycolysis	62
miRNA	miR-598-3p	RMP, IGF1r	Downregulation promotes glycolysis by increasing expression of RMP and IGF1r	Promotes glycolysis, supports gastric cancer cell growth and proliferation	63
miRNA	miR-BART6-5p	SMAD4	Targets SMAD4, activating TGF-β signaling, enhancing glycolytic activity	Enhances glycolysis, promotes cell survival and proliferation	64
miRNA	miR-148b-5p	ATPIF1	Targets ATPIF1, regulates mitochondrial metabolism and glycolysis	Modulates mitochondrial metabolism, enhancing glycolysis	65
miRNA	miR-654-3p	LATS2	Targets LATS2, inhibits glycolytic flux	Inhibits glycolysis, enhances cancer cell death and therapeutic sensitivity	7
miRNA	miR-34a	LDHA	Directly suppresses LDHA expression, influencing glycolysis-dependent resistance	Inhibits glycolysis, increases chemotherapy sensitivity	6
miRNA	miR-513a-3p	HK2	Inhibits HK2 expression, suppressing glycolysis	Reduces glycolysis, slows gastric cancer cell proliferation	66
miRNA	miR-4500	STAT3	Targets STAT3, promoting glycolysis pathways	Enhances glycolysis, promotes tumor cell growth and migration	44

#### 2.3.1 Long non-coding RNAs

Long non-coding RNAs (lncRNAs) regulate glycolysis in gastric cancer. Studies have shown that LINC00242 promotes glycolysis and tumor cell proliferation via the miR-1-3p/G6PD axis. Specifically, LINC00242 is highly expressed in gastric cancer tissues and cells and positively correlates with G6PD expression. Silencing LINC00242 significantly inhibits both proliferation and glycolysis, likely through competitive binding to miR-1-3p, which normally suppresses G6PD expression, thereby affecting tumor metabolism and growth (42).

Additionally, lncRNA SNHG7 modulates glycolysis-related chemoresistance by repressing miR-34a, thereby regulating LDHA expression. SNHG7 is significantly upregulated in gastric cancer and is associated with cisplatin resistance. Its knockdown markedly increases the sensitivity of resistant cells to cisplatin, indicating its critical role in drug resistance. Mechanistically, SNHG7 suppresses miR-34a to elevate LDHA expression, promoting glycolysis and tumor cell survival (6).

ABHD11-AS1 promotes gastric cancer progression by enhancing glycolysis through upregulation of c-Myc. It is overexpressed in gastric cancer cells, and its overexpression stimulates both cell proliferation and metabolic activity (55). As a central regulator, c-Myc mediates ABHD11-AS1-driven glycolysis and cell growth.

In contrast, CRYM-AS1 exerts a negative regulatory effect. It binds to EZH2 to control the methylation status of CRYM, thereby suppressing glycolysis and tumor cell proliferation. CRYM-AS1 is downregulated in gastric cancer tissues, and its low expression correlates with poor prognosis. Overexpression of CRYM-AS1 significantly reduces glycolytic activity and proliferation, suggesting its potential as a tumor suppressor and therapeutic target (43).

Together, these findings highlight the diverse and critical functions of lncRNAs in the regulation of glycolysis in gastric cancer, offering new potential targets for metabolic intervention.

#### 2.3.2 Circular RNAs

Circular RNAs (circRNAs), a class of covalently closed non-coding RNAs with enhanced stability, have garnered increasing attention in gastric cancer research (67). Studies show that circRNAs contribute to tumorigenesis and metabolic regulation—particularly glycolysis—primarily by acting as miRNA sponges (68).

For example, circBFAR promotes glycolysis by sponging miR-513a-3p, thereby lifting its repression of HK2 (66). circRPS19 enhances HK2 enzymatic activity by stabilizing the protein via the miR-125a-5p/USP7 axis (57). Similarly, circZNF131 upregulates PFKFB2 expression through miR-186-5p (58), and circUBE2Q2 activates STAT3 signaling by sequestering miR-370-3p, promoting transcription of glycolysis-related genes such as LDHA (59). Moreover, circ_0006089 increases GLUT1 expression through the miR-361-3p/TGFB1 axis (60). Collectively, these mechanisms enhance glucose uptake, lactate production, and ATP generation, driving the malignant progression of gastric cancer.

#### 2.3.3 MicroRNAs

MicroRNAs regulate glycolysis in gastric cancer by directly or indirectly targeting key downstream genes. For instance, miR-181b binds the 3′-UTR of HK2 to inhibit its expression, reducing glucose uptake and lactate production (36). miR-let-7a suppresses glycolytic flux by targeting PKM2 (61), while miR-139-5p targets AMPKα1 (PRKAA1) to influence energy metabolism (62). Downregulation of miR-598-3p leads to increased expression of its targets RMP and IGF1R, promoting glycolysis (63). EBV-associated miR-BART6-5p enhances glycolytic activity via activation of the TGF-β pathway through SMAD4 targeting (64).

Other mechanisms include miR-148b-5p targeting ATPIF1 to modulate mitochondrial metabolism (65), miR-654-3p inhibiting glycolysis via LATS2 (7), and miR-34a directly suppressing LDHA to affect chemoresistance (6). miR-513a-3p inhibits HK2 expression (66), while miR-4500 targets STAT3 to modulate the glycolytic pathway (44). These regulatory events collectively shape the metabolic reprogramming of gastric cancer cells and highlight the central role of miRNAs in controlling glycolysis.

### 2.4 Tumor microenvironment and exosomes

In the gastric cancer microenvironment, tumor-associated macrophages (TAMs) are crucial to disease pathogenesis. TAMs and their derived exosomes drive glycolytic reprogramming by modulating multiple downstream signaling pathways. For example, exosomes derived from M2-polarized TAMs deliver MALAT1 into gastric cancer cells, which promotes glycolysis through dual mechanisms: it blocks the ubiquitin-mediated degradation of β-catenin (by inhibiting β-TRCP), thereby activating the Wnt pathway; and it acts as a molecular sponge for miR-217-5p, lifting suppression of HIF-1α expression. These actions synergistically upregulate key glycolytic enzymes and increase lactate production (69).

Conversely, gastric cancer cells secrete PKM2-enriched exosomes that are taken up by TAMs, inducing their polarization toward the M2 phenotype and activating the PI3K/Akt/mTOR axis in a feedback loop. This signaling cascade enhances glycolytic flux by upregulating hexokinase (HK2) and glucose transporter (GLUT1) expression (21). Additionally, exosomal lncMMPA competitively binds miR-548s to upregulate ALDH1A3, further strengthening the coupling between glycolysis and the tricarboxylic acid (TCA) cycle (70). Moreover, glycolytic byproducts such as lactate stabilize IGF2BP3 via circSIPA1L3 carried in exosomes, increasing the mRNA stability of lactate transporter SLC16A1 and forming a positive feedback loop to sustain glycolysis (71).

Interventional studies have confirmed the therapeutic relevance of this network. Silencing exosomal MALAT1 significantly inhibits tumor growth in mice and reverses chemoresistance (69). Similarly, the traditional Chinese herbal formula mJPYZ reduces exosomal PKM2 levels, disrupting TAM M2 polarization and glycolysis-dependent tumor progression (21).

The metabolic reprogramming mediated by M2 macrophages and their exosomes emphasizes the important role of immune cells in tumor progression and opens new avenues for therapeutic intervention. Further exploration of M2 TAMs and their exosomal components in gastric cancer may uncover novel mechanisms and targets for disrupting the tumor-supportive microenvironment.

### 2.5 Hypoxia and metabolism

Hypoxia is a hallmark of the tumor microenvironment and significantly contributes to the progression of gastric cancer (As shown in **Table 4**). It alters the metabolic profile of tumor cells by shifting energy production from oxidative phosphorylation to aerobic glycolysis, a phenomenon known as the Warburg effect (72). Under hypoxic conditions, this metabolic shift is primarily driven by the activation of key signaling pathways and molecular regulators—most notably hypoxia-inducible factor-1 alpha (HIF-1α). When oxygen levels are low, HIF-1α upregulates the expression of glycolytic enzymes such as hexokinase 2 (HK2), lactate dehydrogenase A (LDHA), and phosphofructokinase 1 (PFK1), which increases glucose uptake and lactate production, thereby enhancing glycolytic flux (73).

**Table 4:** Exosome and tumor microenvironment regulating glycolysis.

Exosome source	Main molecule	Target genes/pathways	Mechanism of action	Functional impact	References
Tumor-Associated Macrophages (TAM) Exosomes	MALAT1	Wnt, β-catenin, HIF-1α	MALAT1 blocks β-catenin ubiquitination degradation, activating the Wnt pathway, while adsorbing miR-217-5p to relieve the suppression of HIF-1α, promoting the expression of glycolytic enzymes	Promotes glycolysis, enhances lactate production, supports tumor cell proliferation and growth	69
Gastric Cancer Cell Exosomes	PKM2	PI3K/Akt/mTOR, HK2, GLUT1	Exosomal PKM2 is delivered to TAM, inducing M2 polarization, activating the PI3K/Akt/mTOR axis, upregulating HK2 and GLUT1 expression, and enhancing glycolytic flux	Enhances glycolytic flux, promotes tumor growth and drug resistance	21
Gastric Cancer Cell Exosomes	circSIPA1L3	IGF2BP3, SLC16A1	circSIPA1L3 stabilizes IGF2BP3, increasing mRNA stability of SLC16A1, forming a positive feedback loop	Supports sustained glycolytic flux, promotes tumor proliferation and metastasis	71
Exosomal lncMMPA	lncMMPA	ALDH1A3	Competes with miR-548s to upregulate ALDH1A3 expression, enhancing glycolysis and TCA cycle coupling	Strengthens glycolysis and energy metabolism, supporting tumor growth	70
Exosomal MALAT1	MALAT1	Not specified	Targeting and silencing exosomal MALAT1 significantly inhibits tumor growth and reverses chemotherapy resistance	Slows tumor progression, restores chemotherapy sensitivity	69

HIF-1α also regulates downstream targets like CCL7 and KIAA1199, forming a HIF-1α/CCL7/KIAA1199 axis that accelerates glycolysis and promotes tumor progression (74). Furthermore, hypoxia alters the expression of several microRNAs. For example, downregulation of miR-598-3p releases its inhibitory effects on RMP and IGF1R, leading to glycolytic activation (63). Similarly, reduced miR-186 expression under hypoxic conditions lifts suppression of HIF-1α, further increasing the expression of glycolytic enzymes such as HK2 (75).

Hypoxia also enhances CD73 activity, which promotes adenosine production, supporting the Warburg effect and reinforcing the metabolic reprogramming in gastric cancer cells (5). At the transcriptional level, HIF-1α regulates the expression of FOXO4, which binds to the LDHA promoter and modulates its activity, affecting the glycolytic rate (76). Feedback mechanisms, such as the N4-acetylcytidine (ac4C)-mediated NAT10/SEPT9/HIF-1α positive feedback loop, stabilize SEPT9 mRNA under hypoxia and amplify HIF-1α signaling, further exacerbating glycolysis dependency (77). Additionally, genes like AAED1 are involved in HIF-1α-mediated glycolytic activation under hypoxia, supporting tumor cell proliferation and survival (78).

In summary, hypoxia-induced metabolic reprogramming in gastric cancer is primarily orchestrated through the HIF-1α axis. Through multi-level molecular regulation, hypoxia enhances glycolytic enzyme activity and metabolic flux, ultimately promoting malignancy and treatment resistance. Understanding this hypoxia-driven metabolic shift provides key insights into tumor biology and offers promising avenues for therapeutic intervention.

## 3 T glycolysis and chemoresistance

### 3.1 Glycolytic mechanisms in resistance

Glycolysis significantly contributes to the development of chemoresistance in gastric cancer (As shown in **Table 5**). One study revealed that the Helicobacter pylori virulence factor CagA induces resistance to 5-fluorouracil (5-FU) by activating the Akt pathway and upregulating glycolysis. Specifically, CagA expression is significantly elevated in 5-FU–resistant gastric cancer cells, along with increased expression of glycolytic enzymes such as hexokinase 2 (HK2) and lactate dehydrogenase A (LDHA). This enhanced glycolytic activity supports cancer cell survival. Inhibition of glycolysis or the Akt pathway effectively reverses this resistance and restores 5-FU cytotoxicity, providing new insight into therapeutic strategies (54).

**Table 5:** Hypoxia and metabolic resistance mechanisms in gastric cancer.

Mechanism/factor	Upstream factors	Downstream targets/effects	Mechanism of action	Functional impact	References
HIF-1α	Hypoxia, ROS	HK2, LDHA, GLUT1	HIF-1α upregulates glycolytic enzymes in hypoxic conditions, enhancing glucose uptake and lactate production	Promotes glycolysis, enhances cell survival and drug resistance	73, 74
miR-598-3p	Hypoxia	RMP, IGF1r	Downregulation of miR-598-3p releases inhibition on RMP and IGF1r, activating the glycolytic pathway	Promotes glycolysis, enhances cancer cell proliferation and energy supply	63
miR-186	Hypoxia	HIF-1α	Downregulation of miR-186 relieves inhibition on HIF-1α, further upregulating the expression of HK2 and other glycolytic enzymes	Enhances glycolytic flux, promotes tumor cell proliferation and growth	75
CD73	Hypoxia	Adenosine	CD73 promotes the generation of adenosine, activating the Warburg effect (aerobic glycolysis)	Maintains glycolytic metabolic reprogramming, promotes tumor cell survival and proliferation	5
FOXO4	HIF-1α	LDHA	HIF-1α regulates FOXO4 expression, which binds to the LDHA promoter to regulate its activity	Affects glycolytic rate, promotes tumor metabolic reprogramming	76
ac4C Feedback Loop	Hypoxia, N4-acetylcytidine (ac4C)	NAT10, SEPT9, HIF-1α	The ac4C-mediated NAT10/SEPT9/HIF-1α positive feedback loop enhances HIF-1α signaling, stabilizing SEPT9 mRNA	Stabilizes HIF-1α signaling, enhancing glycolytic metabolism	77
AAED1	Hypoxia	HIF-1α	AAED1 participates in HIF-1α-mediated glycolysis activation, supporting cell proliferation and survival	Supports cell proliferation and survival, providing support for metabolic reprogramming	78
CagA	Helicobacter pylori Infection	HK2, LDHA, 5-Fu resistance	CagA protein activates glycolysis through the Akt pathway, mediating 5-Fu resistance, and upregulating glycolytic enzymes	Enhances glycolysis, increases tumor cell survival and chemotherapy resistance	54
SNHG7-miR-34a-LDHA Axis	M2 Macrophage Exosomes	GLUT1, LDHA	MALAT1 regulates HIF-1α and β-catenin expression, enhancing glycolysis and promoting cisplatin resistance	Enhances glycolysis and chemotherapy resistance	69
Pyrimidine Synthesis Pathway	CAD, DHODH	c-Myc, GLUT1, HK2	Upregulation of CAD and DHODH activates the Notch signaling pathway, promoting cMyc expression, and increasing glycolytic enzymes	Enhances glycolytic flux, increases chemotherapy resistance	79
Short-Form RON Receptor	AKT/GSK3β	GLUT1, LDHA	sf-RON receptor stabilizes β-catenin via the AKT/GSK3β pathway and activates SIX1, promoting GLUT1 and LDHA transcription	Enhances glycolysis, accelerates tumor proliferation, and reduces chemotherapy sensitivity	80
Mitochondrial Metabolism Defects	Mitochondrial Dysfunction	GRP78	Mitochondrial dysfunction in gastric cancer cells leads to glycolysis dependence, accompanied by overexpression of GRP78	Restoring mitochondrial function reverses glycolytic phenotype, recovers 5-FU sensitivity	81

The SNHG7–miR-34a–LDHA axis has also been implicated in cisplatin resistance. MALAT1, delivered via exosomes from M2-type TAMs, contributes to this process by modulating HIF-1α and β-catenin expression, thereby enhancing glycolytic activity and driving cisplatin resistance. Mechanistically, MALAT1 binds to β-catenin, preventing its ubiquitin-mediated degradation and activating the Wnt/β-catenin pathway. It also sponges miR-217-5p, lifting its suppression of HIF-1α. Together, these mechanisms lead to increased expression of GLUT1 and LDHA, enhancing glycolysis and promoting drug resistance (69).

Activation of *de novo* pyrimidine synthesis is another mechanism linked to chemoresistance. Upregulation of CAD and DHODH activates the Notch signaling pathway and promotes c-Myc expression, which in turn upregulates glycolytic enzymes and strengthens the chemoresistant phenotype. Inhibiting this pathway has been shown to enhance the efficacy of chemotherapeutic agents like 5-FU, suggesting its therapeutic potential (79).

Research on short-form RON (sf-RON) receptors further supports the role of glycolysis in resistance. sf-RON activates the AKT/GSK3β pathway, stabilizing β-catenin and upregulating the transcription factor SIX1, which enhances transcription of GLUT1 and LDHA. This process increases glucose uptake and lactate production, thereby promoting tumor proliferation and reducing chemosensitivity (80).

Additionally, mitochondrial metabolic defects contribute to 5-FU resistance. Impaired mitochondrial function in gastric cancer cells leads to a dependence on glycolysis and is accompanied by GRP78 overexpression. Mitochondrial transfer from normal gastric epithelial cells reverses GRP78-mediated stemness traits and the glycolytic phenotype, restoring sensitivity to 5-FU. This suggests that restoring mitochondrial function may be an effective strategy to overcome chemoresistance (81).

### 3.2 Therapeutic targets

#### 3.2.1 Molecular targets

##### 3.2.1.1 lncRNA MALAT1

lncRNA MALAT1 is crucial in shaping the tumor microenvironment, particularly in regulating tumor-associated macrophages (TAMs) (As shown in **Table 6**). Research has demonstrated that M2-polarized TAMs transfer MALAT1 to tumor cells via exosomes, activating glycolysis. This finding opens a promising avenue for therapy: silencing MALAT1 using exosome-delivered siRNA effectively suppresses tumor progression and metastasis (69). MALAT1’s elevated expression strongly correlates with poor prognosis in gastric cancer, highlighting its potential as a therapeutic target, particularly for RNA interference-based treatments.

**Table 6:** Therapeutic targets and drug interventions in glycolysis of gastric cancer.

Target Type	Target molecule	Target genes/pathways	Mechanism of action	Functional impact	References
Molecular Targets	MALAT1	TAMs, Glycolytic Enzymes	Exosomal transfer of MALAT1 activates glycolysis, promoting tumor cell growth and metastasis	RNA interference therapy target, inhibits tumor growth and metastasis	69
Molecular Targets	miR-598-3p	RMP, IGF1r	Overexpression of miR-598-3p or R406 inhibitor inhibits tumor metastasis	Potential therapeutic target in hypoxia-induced tumor cell metabolic reprogramming	63
Molecular Targets	EBV-miR-BART6-5p	SMAD4, TGF-β Pathway	Targets SMAD4 to activate the TGF-β pathway, enhancing glycolysis and promoting tumor proliferation and migration	Slows tumor progression, inhibits glycolysis, enhances radiotherapy effect	64
Glycolytic Enzyme Targets	PFKFB3	Immune Microenvironment, Glycolysis	PFKFB3 regulates glycolytic flux and immune cell function in the tumor microenvironment, enhancing tumor cell survival	Prognostic biomarker and therapeutic target	82
Glycolytic Enzyme Targets	ALDOB	Glycolysis, Tumor Suppression	Restoring ALDOB expression enhances chemotherapy sensitivity	Increases chemotherapy sensitivity, inhibits tumor progression	83
Glycolytic Enzyme Targets	PDK4	Glycolysis, Tumor Metastasis	PDK4 overexpression promotes tumor metastasis, inhibition prevents metastasis	Prevents metastasis, provides new treatment directions	84
Glycolytic Enzyme Targets	HSP90	Glycolytic Enzyme Complexes	HSP90 mediates the assembly of glycolytic enzyme complexes, promoting tumor drug resistance	Reverses drug resistance, enhances therapeutic efficacy	30
Signaling Pathway Targets	HIF-1α	IGF2BP2, m6A Modification	Targeting HIF-1α to enhance radiotherapy and immunotherapy effects	Enhances effects of radiotherapy and immunotherapy	85
Signaling Pathway Targets	SET, CIP2A	c-Myc, Glycolytic Enzymes	SET and CIP2A inhibit PP2A, enhancing c-Myc stability, promoting glycolysis and tumor growth	Promotes glycolysis and tumor metabolic reprogramming	50
Dual Metabolic Inhibition	OXPHOS + Glycolysis Inhibition	Glycolysis, Oxidative Phosphorylation	Simultaneously inhibits oxidative phosphorylation and glycolysis, reducing tumor cell energy supply	Enhances anti-tumor effects, reverses metabolic imbalance	86
Natural Compound Targets	Salidroside	ENO1, PKM2, GLUT1	Downregulates ENO1, PKM2, and GLUT1 expression to inhibit glycolysis	Inhibits glycolysis, induces gastric cancer cell apoptosis	87
Natural Compound Targets	WZ35	ROS, YAP, JNK	Activates ROS-YAP-JNK signaling pathway to inhibit glycolysis	Anti-tumor activity, inhibits gastric cancer cell metabolism and proliferation	88
Natural Compound Targets	EGCG	PI3K/Akt, HIF-1α, LDH	Inhibits PI3K/Akt signaling and reduces HIF-1α and LDH expression, blocking glycolysis	Inhibits aerobic glycolysis, inhibits gastric cancer cell growth	89
Natural Compound Targets	Resveratrol	c-Myc, p53	Regulates c-Myc and p53 signaling pathways, reduces HK2 expression and lactate production, inhibiting glycolysis	Inhibits glycolysis, suppresses tumor growth	90
Natural Compound Targets	Quercetin	c-Myc, HIF-1α	Inhibits HIF-1α and glycolytic enzymes by regulating c-Myc signaling	Inhibits glycolysis, reduces tumor cell metabolic activity	91
Small Molecule Targets	CD73	Glycolytic Enzymes	CD73 inhibitor APCP significantly inhibits tumor growth	Therapeutic target, intervenes in glycolytic pathways	5
Small Molecule Targets	2-DG	Glycolysis	2-DG inhibits glycolysis, reducing cancer cell energy supply, suppressing proliferation and metastasis	Enhances treatment efficacy, overcomes resistance	92
Small Molecule Targets	Ciclopirox Olamine (CPX)	SOX2, HIF-1α, c-MYC	Activates SOX2, HIF-1α, and c-MYC transcription factors, inducing glycolysis and cell metabolic reprogramming	Promotes glycolysis, induces cell senescence and anti-tumor effects	93
Small Molecule Targets	PFK15	PFKFB3	PFK15 inhibits PFKFB3, reducing glycolytic flux, inducing G0/G1 arrest and apoptosis	Inhibits gastric cancer cell invasion, suppresses tumor progression	94
Small Molecule Targets	Thioridazine	Akt/mTOR/GLUT1	Inhibits Akt/mTOR/GLUT1 signaling axis, reducing glucose uptake and glycolysis, reversing trastuzumab resistance	Reverses trastuzumab resistance, enhances therapeutic efficacy	95

##### 3.2.1.2 miR-598-3p regulation

miR-598-3p is significantly reduced in hypoxic conditions, resulting in the upregulation of RMP and IGF1R, which in turn promote glycolysis. Restoring miR-598-3p expression or inhibiting RMP with the R406 inhibitor effectively inhibits tumor metastasis, indicating that this pathway offers a promising target for therapeutic intervention in hypoxia-driven metabolic reprogramming (63).

##### 3.2.1.3 EBV

EBV-miR-BART6-5p is overexpressed in Epstein–Barr virus (EBV)-associated gastric cancer, where it targets SMAD4 to activate the TGF-β signaling pathway. This activation promotes both cancer cell proliferation and migration, while also enhancing glycolysis. Targeting EBV-miR-BART6-5p offers a potential novel therapeutic approach, particularly for EBV-associated gastric tumors. Inhibiting this miRNA reduces glycolytic activity and slows tumor progression (64).

#### 3.2.2 Enzyme targets

##### 3.2.2.1 PFKFB3

PFKFB3, a key glycolytic enzyme, is closely linked to patient prognosis in gastric cancer. Studies using machine learning algorithms have uncovered its involvement in shaping the immune microenvironment. Overexpression of PFKFB3 not only drives glycolysis but also modifies immune cell behavior within the tumor microenvironment. It may function as both a prognostic biomarker and a therapeutic target (82).

##### 3.2.2.2 ALDOB

ALDOB functions as a tumor suppressor, and its loss increases glycolysis while correlating with poor prognosis. Restoring ALDOB expression enhances the sensitivity of gastric cancer cells to chemotherapy, underscoring its potential as a therapeutic target. ALDOB’s regulation of glycolytic flux offers a new perspective for targeted treatment strategies (83).

##### 3.2.2.3 PDK4

PDK4 has emerged as a critical glycolytic regulator. Overexpression of PDK4, induced by TOP1MT deficiency, drives tumor metastasis. The PDK4 inhibitor M77976 effectively diminishes metastatic potential, emphasizing the clinical relevance of PDK4 as a therapeutic target for advanced gastric cancer (84).

##### 3.2.2.4 HSP90

HSP90, a molecular chaperone, supports the assembly of key glycolytic enzymes such as PGK1, PKM2, ENO1, and LDHA into functional complexes. This promotes glycolysis and contributes to chemoresistance. Inhibiting HSP90 may disrupt these enzyme complexes, reverse resistance, and enhance the efficacy of anticancer treatments (30).

#### 3.2.3 Signaling targets

##### 3.2.3.1 HIF-1α

HIF-1α is central to metabolic reprogramming and enhances mRNA stability of IGF2BP2 through m6A modification. Targeting HIF-1α could improve the efficacy of radiotherapy and immunotherapy in gastric cancer, providing new therapeutic possibilities (85).

##### 3.2.3.2 SET/CIP2A–PP2A–c-Myc

SET and CIP2A are overexpressed in gastric cancer and inhibit PP2A activity. This causes increased phosphorylation of c-Myc at Thr58, stabilizing c-Myc and boosting glycolytic flux. The SET/CIP2A–PP2A–c-Myc axis maintains metabolic reprogramming and offers a potential target for intervention (50).

#### 3.2.4 Dual inhibition

##### 3.2.4.1 Real-time metabolic monitoring

Dual inhibition of oxidative phosphorylation (OXPHOS) and glycolysis results in synergistic antitumor effects. Real-time metabolic monitoring using 13C-MRSI technology has demonstrated that this combined approach induces energy depletion, metabolic imbalance, and boosts tumor suppression. This strategy offers a new direction for treating gastric and other malignancies, particularly in overcoming resistance and improving therapeutic efficacy (86).

## 4 Natural compounds and small molecules in glycolysis intervention

### 4.1 Plant-derived compounds

Plant-derived compounds have demonstrated significant potential in inhibiting glycolysis in gastric cancer. For instance, salidroside, a component from Rhodiola rosea, inhibits glycolysis by downregulating the expression of ENO1, PKM2, and GLUT1, leading to the induction of apoptosis in gastric cancer cells (87). This suggests that salidroside modulates metabolic pathways, offering a new direction for gastric cancer treatment.

Another compound, WZ35, a curcumin analog, suppresses glycolysis through the ROS-YAP-JNK signaling pathway, exhibiting anti-gastric cancer activity (88). This mechanism expands the understanding of the anticancer activity of plant compounds and emphasizes their potential in modulating oxidative stress and related signaling pathways to inhibit tumor cell proliferation and metabolism.

Green tea catechins, particularly EGCG, also show inhibitory effects on glycolysis in gastric cancer cells. EGCG lowers the expression of HIF-1α and lactate dehydrogenase (LDH) by inhibiting the PI3K/Akt signaling pathway, thus blocking aerobic glycolysis. This property positions EGCG as a promising therapeutic agent in gastric cancer, further reinforcing the concept of targeting glycolysis as a viable treatment strategy (89).

Resveratrol, a compound derived from grapes, modulates glycolysis by regulating the c-Myc and p53 signaling pathways. Resveratrol reduces HK2 expression and lactate production, effectively inhibiting glycolysis and tumor growth in gastric cancer cells. This discovery highlights resveratrol’s potential as an anticancer drug, particularly in targeting metabolic reprogramming (90).

Quercetin, a flavonoid found in various fruits and vegetables, inhibits glycolytic enzyme activity through the c-Myc signaling pathway. It suppresses the expression of HIF-1α and other glycolytic enzymes, thus reducing glycolysis in gastric cancer cells. This intervention demonstrates quercetin’s potential in cancer treatment by targeting the metabolic activity of tumor cells (91).

These studies show that plant-derived compounds can modulate multiple signaling pathways and metabolic processes, providing promising alternatives for gastric cancer treatment by targeting glycolysis and promoting cell apoptosis.

### 4.2 Small molecule inhibitors

Small molecule inhibitors and enzyme activity modulation have emerged as important therapeutic strategies in gastric cancer. CD73, an enzyme closely linked to glycolysis, has garnered attention in gastric cancer research. Inhibition of CD73 activity by APCP significantly suppresses tumor growth, highlighting the importance of targeting glycolytic enzymes for therapeutic development (5). This finding not only provides new insights into gastric cancer treatment but also reinforces the role of CD73 in tumor metabolism.

In addition, 2-deoxy-D-glucose (2-DG) and other glycolytic inhibitors have shown promising results in synergistic anti-tumor effects. 2-DG inhibits glycolysis and reduces the energy supply to cancer cells, thus suppressing their proliferation and metastatic potential. These findings suggest that combining glycolytic inhibitors with other treatment modalities may significantly improve therapeutic outcomes and overcome resistance (92).

Ciclopirox Olamine (CPX), a small molecule inhibitor, has also attracted interest in gastric cancer research. CPX induces metabolic reprogramming by activating transcription factors such as SOX2, HIF-1α, and c-Myc, thereby reprogramming non-cancer stem cells into cancer stem-like cells. This process not only enhances glycolysis but also induces cellular senescence, making CPX a promising agent for the treatment of gastric cancer (93).

PFK15, a small molecule inhibitor targeting the glycolytic rate-limiting enzyme PFKFB3, has demonstrated effectiveness in gastric cancer. PFK15 disrupts glycolytic flux, causing G0/G1 phase cell cycle arrest and apoptosis while inhibiting tumor cell invasion. This inhibition of PFKFB3 further supports its role as a critical target in the development of new anticancer drugs (94).

Thioridazine, another small molecule, downregulates Skp2 expression and inhibits the Akt/mTOR/GLUT1 signaling axis, reducing glucose uptake and glycolysis. This reversal of trastuzumab resistance suggests thioridazine’s potential in overcoming drug resistance in gastric cancer through metabolic reprogramming (95).

These findings highlight the potential of small molecule inhibitors and enzyme modulators in targeting glycolysis for gastric cancer therapy, offering novel therapeutic strategies for overcoming resistance and improving treatment efficacy.

### 4.3 Glycolysis-related gene prognostic value

#### 4.3.1 Gene expression and clinical relevance

PFKFB3, a glycolytic enzyme, is highly expressed in various cancers, including gastric cancer, and its expression correlates with poor prognosis. Studies indicate that overexpression of PFKFB3 drives metabolic reprogramming and supports cancer cell proliferation and survival. Clinically, elevated PFKFB3 expression is associated with advanced tumor stages, tumor size, and lymph node metastasis, suggesting its potential as a prognostic biomarker. Monitoring PFKFB3 expression may assist clinicians in assessing gastric cancer patient prognosis and tailoring treatment strategies (96). Inhibition of PFKFB3 can reduce glycolytic activity and suppress tumor growth, making it a promising therapeutic target in gastric cancer treatment.

ALDOB (fructose-1,6-bisphosphate aldolase B) is involved in glycolysis and is significantly downregulated in gastric cancer tissues compared to normal tissues. This reduced expression is associated with increased tumor invasiveness and metastasis. Restoring ALDOB expression improves the sensitivity of gastric cancer cells to various anticancer agents, particularly chemotherapies like talazoparib and FTI-277. By delaying glycolysis, ALDOB reduces the energy supply, thus inhibiting tumor growth. This mechanism provides a new biomarker for prognosis and supports therapeutic interventions targeting glycolysis (83).

#### 4.3.2 Multi-omics and machine learning

Multi-omics and machine learning approaches have gained increasing importance in glycolysis-related gastric cancer research. These methods enable the identification of key glycolytic biomarkers and provide insights into their relationship with immune regulation, supporting personalized treatment strategies. Recent studies using machine learning algorithms have identified key genes such as PLOD2, CHSY3, SLC2A3, and SLC5A1 that are associated with glycolysis and prognosis in gastric cancer. These biomarkers help differentiate high- and low-risk patients and are linked to tumor progression and immune suppression. Specifically, PLOD2 regulates the PI3K/Akt/mTOR signaling pathway, which enhances glycolysis and tumor progression. The integration of multi-omics data with machine learning techniques offers a powerful tool for personalized treatment and targeted therapy in gastric cancer (97).

Furthermore, bioinformatics tools such as weighted gene co-expression network analysis (WGCNA) are valuable for uncovering the complex interactions between glycolytic genes and the gastric cancer microenvironment. WGCNA identifies gene modules associated with specific biological processes, providing deeper insights into how glycolysis impacts gastric cancer biology. One study revealed that different glycolytic subtypes correlate with gastric cancer prognosis and immune response, indicating that glycolytic patterns may influence treatment responses and patient outcomes (98).

These advancements in multi-omics and machine learning methodologies are transforming our understanding of glycolysis in gastric cancer and offer promising pathways for personalized medicine and targeted therapy.

## 5 Future directions

The study of glycolysis in gastric cancer has provided significant insights into its metabolic reprogramming and its vital role in tumor progression, chemoresistance, and immune modulation. However, substantial challenges and opportunities remain for future research, particularly in translating these findings into effective therapeutic strategies.

### 5.1 Targeting glycolysis in therapy

Although inhibiting glycolytic enzymes and metabolic reprogramming has shown promising preclinical results, translating these findings into clinical practice presents ongoing challenges. Many glycolytic inhibitors, such as 2-deoxy-D-glucose (2-DG) and PFK15, have demonstrated antitumor effects *in vitro* and in animal models but encounter obstacles in clinical trials, including off-target effects, limited bioavailability, and poor tissue penetration. To address these issues, the development of more selective and potent inhibitors, alongside innovative drug delivery systems (e.g., nanoparticles or exosome-based therapies), is essential. Tailored approaches that take into account individual tumor metabolic profiles and specific genetic alterations in glycolysis-related pathways will also be critical in improving treatment efficacy.

### 5.2 Combination therapies

Given the significant involvement of glycolysis in mediating chemoresistance, future research should focus on combination therapies that incorporate glycolytic inhibitors with traditional chemotherapy, immunotherapy, and targeted therapies. Combining glycolysis inhibitors with immune checkpoint inhibitors, such as PD-1/PD-L1 inhibitors, offers the potential to overcome immune evasion and restore antitumor immune responses in gastric cancer. Additionally, pairing glycolytic inhibitors with targeted therapies targeting key signaling pathways (e.g., PI3K/Akt/mTOR) could improve treatment outcomes by disrupting both tumor metabolism and essential survival signals.

Identifying specific biomarkers linked to glycolytic reprogramming will guide the selection of patients most likely to benefit from these combination therapies. Furthermore, biomarkers predicting responses to glycolysis-targeting therapies must be validated in clinical settings to enhance the personalization of treatment.

### 5.3 Tumor microenvironment and metabolic interactions

The tumor microenvironment (TME) influences tumor metabolism and therapeutic response. The interactions between tumor cells, immune cells, stromal cells, and the extracellular matrix create a dynamic metabolic network that supports cancer cell survival and contributes to therapeutic resistance. Future research should focus on gaining a deeper understanding of the metabolic interactions between various TME components, particularly the crosstalk between tumor cells and immune cells such as tumor-associated macrophages (TAMs).

Exosome-mediated metabolic reprogramming presents another promising research direction. Exosomes from tumor cells and TAMs carry molecular cargo (e.g., lncRNAs, circRNAs, proteins, lipids) that influence glycolysis and drive tumor progression in recipient cells. Exploring the role of exosomes in mediating metabolic shifts and their potential use as therapeutic delivery vehicles could open new avenues for targeting metabolic reprogramming in gastric cancer.

### 5.4 Precision medicine in glycolysis therapy

Advances in understanding the genetic and epigenetic alterations driving glycolysis in gastric cancer highlight the growing importance of precision medicine. Genetic profiling and multi-omics technologies enable the identification of glycolytic signatures and the development of personalized treatments. The integration of these biomarkers with advanced imaging techniques, such as positron emission tomography (PET), may aid in real-time monitoring of tumor metabolism and response to glycolysis-targeting therapies.

Moreover, the use of artificial intelligence (AI) and machine learning to analyze large-scale genomic and clinical datasets offers potential for identifying novel glycolytic biomarkers, predicting treatment responses, and improving patient outcomes. By incorporating AI-driven tools into clinical decision-making, the selection of patients for glycolysis-targeting therapies can be optimized, leading to enhanced therapeutic efficacy.

### 5.5 Conclusion

Targeting glycolysis in gastric cancer offers a promising therapeutic strategy that could enhance treatment outcomes and help overcome resistance to conventional therapies. However, significant challenges remain in translating these findings into clinical practice. Future research should focus on developing more effective and selective glycolytic inhibitors, exploring combination therapies, and utilizing precision medicine approaches to personalize treatment. By deepening the understanding of the intricate metabolic networks driving gastric cancer progression, new avenues for therapeutic intervention could be opened, leading to improved prognosis for gastric cancer patients.
